# Ancient DNA reveals phenological diversity of Coast Salish herring harvests over multiple centuries

**DOI:** 10.1038/s41598-022-17656-4

**Published:** 2022-08-06

**Authors:** Eleni L. Petrou, Robert Kopperl, Dana Lepofsky, Antonia T. Rodrigues, Dongya Yang, Madonna L. Moss, Camilla F. Speller, Lorenz Hauser

**Affiliations:** 1 School of Aquatic and Fishery Sciences, University of Washington, 1122 NE Boat Street, Seattle, WA 98105 USA; 2 Willamette Cultural Resources Associates Ltd., 655 S. Orcas St., Ste. 220, Seattle, WA 98108 USA; 3 Department of Archaeology, Simon Fraser University, Education Building 9635, 8888 University Dr., Burnaby, BC V5A 1S6 Canada; 4 Department of Anthropology, University of Oregon, Eugene, OR 97403 USA; 5 Department of Anthropology, University of British Columbia, Vancouver, BC V6T 1Z1 Canada

**Keywords:** Ecology, Genetics, Environmental sciences, Environmental social sciences

## Abstract

Phenological diversity in food resources prolongs foraging opportunities for consumers and buffers them against environmental disturbances. Such diversity is particularly important in forage fish such as Pacific herring (*Clupea pallasii*), which are foundational to coastal food webs and fisheries. While the importance of phenological diversity is well-known from contemporary studies, the extent to which different populations contribute to fisheries over long time scales is mostly unknown. In this study, we investigated the relative contributions of genetically and phenologically distinct herring populations to Indigenous Peoples’ food systems over multiple centuries, using ancient DNA extracted from archaeological herring bones. These bones were excavated from two Coast Salish archaeological sites (Burton Acres Shell Midden and Bay Street Shell Midden) in the Puget Sound region, USA. Using genetic stock identification from seven nuclear DNA markers, we showed that catches at the two sites in central Puget Sound were dominated by January–February and March–April spawners, which are the contemporary spawning groups in the vicinity of the sites. However, May spawners were detected in the older Burton Acres assemblage (dated to 910–685 cal BP), and a mixed stock analysis indicated that catches at this site consisted of multiple populations. These results suggest that Coast Salish ancestors used a portfolio of herring populations and benefited from the ecological resource wave created by different spawning groups of herring. This study of ancient DNA allowed us to glimpse into Indigenous traditional food and management systems, and it enabled us to investigate long-term patterns of biodiversity in an ecologically important forage fish species.

## Introduction

There is growing recognition that phenotypic and genetic diversity contribute to the productivity and resilience of wild populations and the ecosystem goods that they provide^1^. For example, diverse life-histories in salmonids are associated with asynchronous population dynamics which stabilize overall abundance through portfolio effects^2,3^. Meanwhile, intraspecific genetic variation represents the evolutionary potential of populations to adapt to new environmental conditions^4,5^ and develop resistance to pathogens^6,7^. Despite the recognized value of intraspecific variation, population diversity is eroding at a rapid rate globally due to human activities^8,9^. This erosion of diversity has implications for ecosystem health, and in particular for Indigenous Peoples who have relied on this diversity for millennia; for them, the loss of diversity is linked tightly to multiple dimensions of social well-being^10^.

Population and phenological diversity are particularly important in species central to ecosystem functioning that support human societies. Pacific herring (*Clupea pallasii*) are an abundant forage fish in coastal waters of the Pacific Northwest and important prey for a variety of organisms, including marine mammals^11,12^, salmon^13^, and sea birds^14,15^. The spawning migrations of herring provide an ephemeral pulse of food for coastal marine organisms^16^, as fish aggregate to nearshore environments to spawn.

**Figure 1:**
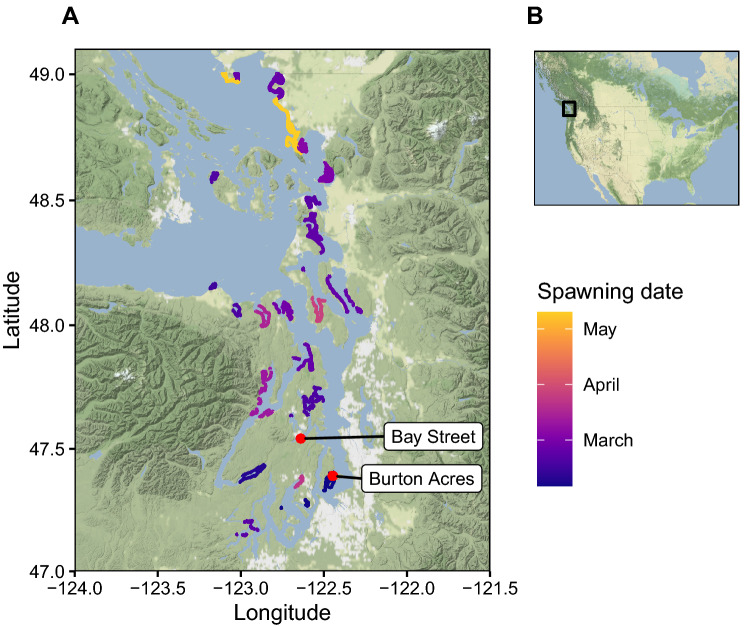
Map of archaeological sites (red points) in relation to contemporary herring spawning locations in Puget Sound. Spawning locations in Washington State are depicted by lines whose color represents the average spawning date, estimated using multidecadal spawn survey data^17^.

The timing of herring reproduction varies across broad latitudinal gradients, with southern populations typically spawning in winter and northerly populations reproducing in spring. However some geographic areas, for example, the Puget Sound in Washington State, are used by a variety of spawning groups with distinct reproductive timing^17^. Contemporary populations of herring in the Puget Sound have characteristic spawn periods that range from January to May (Fig. 1). Herring with distinct spawn times are genetically differentiated from each other^18^, and can be assigned to specific spawning groups with genetic markers^19^. This diversity in spawn timing creates a resource wave that gives consumers the opportunity to access herring in the nearshore environment over an extended time^20,21^.

The resource wave created by herring may have been important to the livelihoods of human communities on the Pacific coast of North America. Humans harvested herring for millennia^22^, as demonstrated by specimens found in archaeological sites in Southeast Alaska that are dated to ~ 10,000 years before present^23^. Throughout the Pacific Northwest, herring bones are among the most abundant fish remains found in ancient coastal settlements^22,24,25^, indicating that herring were a critical food resource. Artifacts such as herring rakes and fish traps^26^ attest to past harvesting techniques, and early ethnographic studies contain lively descriptions of their use. Accounts from the late 1800s indicate that herring were eaten fresh or cured (e.g., by the Skokomish, Klallam, and Twana;^27^), while herring eggs were harvested using evergreen branches or kelp fronds that were submerged during spawning events (e.g., by the Puyallup and Nisqually;^28^). Today, herring eggs harvested in this way are a beloved food item in many communities (e.g., by the Tlingit and Haida;^29^) and herring harvest is an expression of Indigenous culture and sovereignty (e.g., by the Heiltsuk;^30–32^), while income generated from herring fisheries supports coastal livelihoods^30^.

The contemporary use of herring by Indigenous People may be affected by reduced phenological diversity following overexploitation in industrial fisheries. Industrial commercial fisheries for herring began with the colonization of the Pacific Northwest coast in the late nineteenth century by European settlers, and declining catches were reported as early as 1886 in the Puget Sound^33^. In the twentieth century, large reduction and sac-roe fisheries harvested thousands of tons of herring yearly, but collapsing herring populations in Washington State led to their closure in the latter part of the century^34^. Today, Washington State only allows a bait fishery to operate in the Puget Sound^17^.

Despite the three decades since the last large commercial herring fisheries operated in Puget Sound^35^, an approximately 50-year time series of spawning biomass surveys indicates that certain herring populations have not recovered to their former abundance and remain severely depressed^17^. For example, the May-spawning population from Cherry Point was once the largest stock in Puget Sound, but this population has declined over 96% from the initial biomass estimate made in 1973^17^. It is unclear if the dramatic decline of this population is representative of natural variation in population productivity or if this pattern is unique to recent decades, reflecting anthropogenic impacts.

One method to gather ecological data over long time scales is to quantify faunal remains in paleontological or archaeological deposits. Although coarser in temporal resolution than modern ecological studies^36^, these data can supply long-term information about ecological conditions^37,38^ and patterns of resource variability^39,40^. Additional layers of information can be provided by analyzing ancient DNA (aDNA) preserved in archaeological remains or sediments, as these archive the genetic diversity of wild food resources that sustained human communities over long periods of time. For example, studies of aDNA in Pacific salmon have identified the migration phenotypes that supported ancient fisheries^41,42^ and quantified reductions in genetic diversity associated with the large-scale habitat degradation of riverine habitats in the twentieth century^41^.

In this study, we estimated the relative contributions of genetically distinct populations of Pacific herring to Indigenous food systems over the last millennium, using aDNA from herring bones preserved in archaeological sites in the Puget Sound region. Specifically, we ask the following questions: (i) Which populations of herring did Coast Salish people in the central Puget Sound harvest? (ii) Did the relative proportions of these herring populations vary through time in the archaeological record? (iii) Did the relative proportions of these herring populations differ between archaeological sites? (iv) How does archaeological herring population diversity compare to contemporary population diversity? Answering these questions allowed us to glimpse into Indigenous traditional food and management systems and long-term connections to place and marine resources. Additionally, it allowed us to investigate long-term patterns of biodiversity in an ecologically important forage fish species.

## Methods

### Study system

#### The ecological and cultural context of Coast Salish herring fisheries

The Puget Sound ecosystem is a large fjord estuary fed by freshwater from multiple rivers that flow from the Olympic and Cascade Mountains. The diverse topography of the region supports high levels of biodiversity and productive coastal habitats rich in marine resources, including shellfish, finfish, and marine mammals^43^. Archaeological studies indicate that Indigenous People have lived in the Puget Sound region since at least 12,000 years before present^44^. Today, there are 23 federally recognized Native American Tribes and six tribes that are not federally recognized and/or seeking federal recognition in the Puget Sound region. Some of these tribes are linguistically related, and based on these linguistic relations they are known collectively as the Coast Salish.

Various oral histories and ethnographies document the importance of herring to Coast Salish peoples in the early twentieth century. In the Puget Sound region, both herring and herring eggs (roe) were harvested by Coast Salish tribes including the Nisqually^28^, Puyallup^28^, Klallam^45^, Twana^46^, Skokomish^46^, Suquamish^47^, and Lummi^48^. Herring were harvested from shallow water using baskets, mazes, and fish traps, and from deeper water using rakes and dip nets that were deployed from canoes^49^. Herring roe was considered a delicacy, and it was collected from spawning sites by placing kelp or hemlock branches in the water as herring reproduced^45^. Herring and roe were eaten both fresh and dried^28^, and historically these foods were cured for trade with inland Tribes^49^. Herring were widely used as bait for salmon and also valued as “unharvested bait” that facilitated the harvest of other species—for example, Coast Salish people caught salmon, waterfowl, and seals as they aggregated to prey upon schools of herring^49^.

#### Description of archaeological sites

Ancient herring bones were previously excavated from two archaeological sites in the Puget Sound^50,51^. We received permission from the Puyallup Tribe of Indians to use herring bones retrieved from the Burton Acres Shell Midden (45KI437), and the Suquamish Tribe permitted the use of bones excavated from the Bay Street Shell Midden (45KP115). The bones from 45KI437 are curated at the Burke Museum of Natural History and Culture, and the bones from 45KP115 are curated at the Suquamish Museum. Both archaeological sites are located within protected bays in the Puget Sound that encompass or are close to (within 10 km) contemporary herring spawning grounds (Fig. 1), and the sites are separated by a waterway distance of approximately 50 km. Deposits of artifacts, thermally modified rocks, shell fragments, and animal bones have been found at each site, indicating that people caught and processed fish and shellfish, among other activities, at these locations^50,51^.

The Burton Acres Shell Midden is located along the shoreline of Quartermaster Harbor on Vashon Island ^50^, an area in the traditional territory of the Puyallup Tribe of Indians. Archaeological evidence suggests that the site was first occupied approximately 800 years ago and its deposits indicate continuous occupation into the post-European American contact era that began in south Puget Sound in the early nineteenth century. The midden’s uppermost layers contain metal and other materials of European American manufacture, demonstrating the site’s use into the last two centuries^50^. Furthermore, Puyallup oral histories document several villages within short distances from the Burton Acres Shell Midden in the early twentieth century^52^. The large deposits of fish and shellfish found at Burton Acres, as well as the site’s ample wind exposure, indicate that this was an important location for the drying and preservation of marine resources^52^. Herring bones are consistently abundant across archaeological layers at the Burton Acres Shell Midden, indicating a long-term focus on herring. Of the 16 fish taxa identified, Pacific herring are the most abundant, contributing up to 90% (range = 54–92%) of fish remains in certain layers^24^, while salmonids are the next most abundant group.

The Bay Street Shell Midden is located on the coastline of Sinclair Inlet on the Kitsap Peninsula, on the traditional territory of the Suquamish Tribe. Archaeological evidence shows that the site was used over a period of approximately 800 years, and ethnographic records indicate that the site was used seasonally in post-contact times by the Suquamish as they gathered and preserved food^51^. People at this site had access to both riparian and marine resources, and eight different fish taxa were identified at this site. Pacific herring account for one-third of identifiable fish remains in the total assemblage, indicating prominence in the diet (Supplemental Fig. 4). Flatfish account for another one-third, while other taxa occur in smaller proportions (each composing 10% or less of the fish remains) and include salmonids and various nearshore marine fish species.

Two chronological units—one early and one late—were established for both archaeological sites to enable a diachronic comparison of herring genetic diversity. Herring bones (SI Data 1 and SI Data 2) were selected from the oldest and the more recent securely-dated deposits at each site (*N* = 48 bones from each chronological unit). When available, we selected prootic bones for aDNA analysis to avoid sampling the same individual herring multiple times.

At the Burton Acres Shell Midden, archaeological herring specimens from the deepest excavated stratum (2F) were associated with charcoal yielding a calibrated radiocarbon age estimate of 910–685 calibrated years before present (cal BP). Specimens from the stratigraphically more recent Stratum 2B was likely deposited after AD 1890 because this stratum was not intermixed with underlying deposits and it contained metal artifacts (such as a dime minted between AD 1860 and 1890), and an 1890s button^53^. We hereinafter refer to this layer as “post-contact”.

Archaeological herring specimens from the Bay Street Shell Midden were selected from the oldest (Component 1) and the most recent (Component 3) of the three components defined for the site^51^ to provide the greatest diachronic separation of samples. Radiocarbon dates provided an age estimate of 800–550 cal BP for Component 1 and an estimate of 400–100 cal BP for Component 3. Table 1 provides additional data associated with the particular deposits from which the herring bones for aDNA analysis were selected.

**Table 1:** The relative age of each archaeological sample collection.

Site name	Provenience	Sample materials	Lab sample code	Lab date (RYBP)	Est. δ^13^C (%)	Calibrated age (2σ Cal B.P.)^b^	Chronological analytic unit
Burton Acres Shell Midden	Units 28/58 and 29/58, Strat 2B	Historic materials^a^	n/a	n/a	n/a	n/a	Shallow deposits (circa AD 1860–1920)
Burton Acres Shell Midden	Unit 29/58, Strat 2F	Charcoal	Beta-96013	870 ± 50	− 25.0	910–685	Base of deposit^c^ (910–685 cal BP)
Bay Street Shell Midden	Unit 9, Strat B-1, Fea. 2	Charred bark	Beta-122702	120 ± 50	*	280-modern	Component 3 (400–100 cal BP)
Bay Street Shell Midden	Unit 9, Strat B-1	Charred wood	Beta-122704	250 ± 50	*	465-modern	Component 3 (400–100 cal BP)
Bay Street Shell Midden	Unit 7, Strat A-4, Fea. 1	Charcoal	Beta-122701	810 ± 50	*	900–665	Component 1 (800–550 cal BP)
Bay Street Shell Midden	Unit 7, Strat B-1	Charcoal/charred wood aggregate	Beta-122703	750 ± 120	*	915–540	Component 1 (800–550 cal BP)

*Not provided in the original report^51^.

^a^Historic material limited to upper strata (1, 2A-2C); temporally diagnostic items include a Seated Liberty dime, lead shot, and a button.

^b^Calibrated with OxCal 4.4 with IntCal 20^83,84^.

^c^Two other radiocarbon dates were obtained from Strat 2F. One was one on unburned wood that required extended counting and resulted in a lab date of 120 ± 80 RYBP and was interpreted as intrusive into the deposit from more recent activity^53^. The second was on a fragment of marine shell resulting in a lab date of 1000 ± 60 RYBP, and once corrected for marine reservoir effects yielded a calibrated probabilistic age estimate centered on about 800 cal BP^85^.

### DNA extraction of archaeological samples

All sample preparations and aDNA extractions were conducted in a dedicated aDNA laboratory at Simon Fraser University, Canada, following strict protocols to minimize contamination with modern DNA^54^. Bones were chemically decontaminated through immersion in 6% sodium hypochlorite solution for 5 min. Samples were subsequently rinsed twice with ultra-pure water and bones were exposed to UV light for 15 min. We extracted DNA from each sample following the protocol described in Yang et al.^55^. In brief, samples were incubated overnight at 50 °C in 3 mL of lysis buffer (0.5 M EDTA, 0.5 mg/mL Proteinase K), and were subsequently concentrated to approximately 100 µL using Amicon 10kDA centrifugal filter units. DNA extracts were purified using Qiagen MinElute columns following the manufacturer’s instructions, and DNA was eluted in 50 µL of warmed (56 °C) Buffer EB. To test for contamination during DNA extraction, blank controls were included in each extraction and processed during all subsequent steps.

### SNP genotyping of ancient samples

Each archaeological sample was genotyped at seven nuclear SNP loci using custom TaqMan assays. These seven loci had divergent allele frequencies when comparing herring populations that spawn at different times of year in the Puget Sound^19^. We hereinafter refer to these genetically distinct herring populations as January–February spawners, March–April spawners, and May spawners. One of these loci is within a gene (*SYNE2*) which influences the development of retinal photoreceptors in vertebrates^56^ and may play a role in the photoperiodic regulation of reproduction in herring^18,57^. Additional information on assay development is described in Chamberlin et al.^19^.

To increase the amount of template aDNA available for TaqMan genotyping reactions, we first conducted a preamplification PCR with all primers following the protocol of Smith et al.^58^. These reactions were set up in a dedicated clean room at the University of Washington. Preamplification reactions were conducted in 24 µL volumes containing Qiagen Multiplex PCR Master Mix, 0.2 µM of each forward and reverse SNP primer, ultra-pure water, and 4 µL of template aDNA. Thermal cycling was performed on a Bio-Rad C1000 Touch (Hercules, CA), using these conditions: initial denaturation at 95 °C for 15 min, followed by 14 cycles of 94 °C for 30 s, 57 °C for 90 s, 72 °C for 60 s, and a final extension of 72 °C for 10 min. Negative controls were included with each preamplification reaction.

We diluted these preamplification PCR products 1:3 for use in subsequent TaqMan genotyping reactions. All genotyping reactions took place in 12 µL volumes containing 1X TaqMan Universal PCR Master Mix, 1X TaqMan assay, nuclease-free water, and 2 uL of template DNA. Thermal cycling was performed on an Applied Biosystems 7900HT Fast Real-Time PCR system (Foster City, CA) as follows: initial denaturation at 95 °C for 10 min, followed by 60 cycles of 95 °C for 15 s and 60 °C for 60 s. Following quality control procedures established in Speller et al.^59^, each sample was genotyped twice and negative controls were included with every genotyping reaction. We estimated the genotyping discrepancy rate by dividing the total number of genotype mismatches by the total number of samples. Ancient samples that were missing genotypes at more than one locus were removed from the data set*.*

### Comparison of modern and ancient herring

We used restriction site associated DNA sequencing data reported in Petrou et al.^18^ as the modern herring data set. These modern herring samples (N = 347) were collected from eight distinct spawning aggregations in the Puget Sound between 2007 and 2016 (Supplemental Table 1 and Supplemental Fig. 1) and genotyped at 6,718 SNPs. For a full description of how these data were generated, see Petrou et al.^18^. Genotypes at the same seven nuclear loci as the archaeological herring were used for this study.

Pairwise *F*_*ST*_^60^ between ancient and modern herring collections was calculated in *hierfstat*, and its significance was estimated using 1000 permutations in the R package *strataG*^61^. Patterns of genetic differentiation in the ancient and modern samples were visualized using a PCA conducted with the R package *adegenet*^62^. We estimated expected heterozygosity, observed heterozygosity, and *F*_IS_ for each locus in every archaeological layer separately using *hierfstat*. Loci were subsequently tested for deviations from Hardy–Weinberg equilibrium (HWE) using exact tests in *genepop*^63^.

We estimated the contribution of genetically distinct herring populations to ancient food systems by conducting a mixed stock analysis using the Bayesian method described in Moran and Anderson^64^ and implemented in the R package *rubias*^64^. In brief, this approach uses Markov chain Monte Carlo (MCMC) to estimate the proportion of individuals in a mixture originating from different reference populations (or aggregates of reference populations known as reporting groups), given genotypic data in the reference populations. Additional information on how we used simulated and empirical data to assess the predicted accuracy of mixed stock analysis is included in the Supplemental Material. Using the seven nuclear loci and three reporting groups (January–February spawners, March–April spawners, and May spawners), the predicted accuracy of mixed stock analysis ranged from 85 to 88% using simulated data, and 97–99% using empirical samples from contemporary herring populations (Supplemental Fig. 3).

In the mixed stock analysis of archaeological herring, we used modern herring samples as the reference populations and we analyzed ancient herring from distinct archaeological layers as separate mixtures. We conducted the mixed stock analysis (number of MCMC iterations = 10,000, burn-in steps = 1000) using three reporting groups (January–February spawners vs. March–April spawners vs. May spawners).

## Results

We were able to successfully genotype 83% of archaeological samples (*N* = 159) at six or more loci. Negative controls did not amplify in any genotyping reaction, and we did not observe any genotype mismatches between repeated genotyping of ancient samples. The number of genotyped ancient samples at each locus, archaeological site, and archaeological layer ranged from 30 to 46 individuals, while observed heterozygosities ranged from 0 to 0.50 (Supplementary Table 2). The number of tests with statistically significant deviations from HWE (α = 0.05) ranged from one to four per archaeological site and layer, and the most deviations from HWE (*N* = 4) were observed in samples collected from the older Burton Acres archaeological layer (910–685 cal BP). Overall, six out of 42 (14%) tests for linkage disequilibrium were statistically significant (α = 0.05) and all of these occurred in the Burton Acres site (there were three significant tests per archaeological layer).

**Figure 2:**
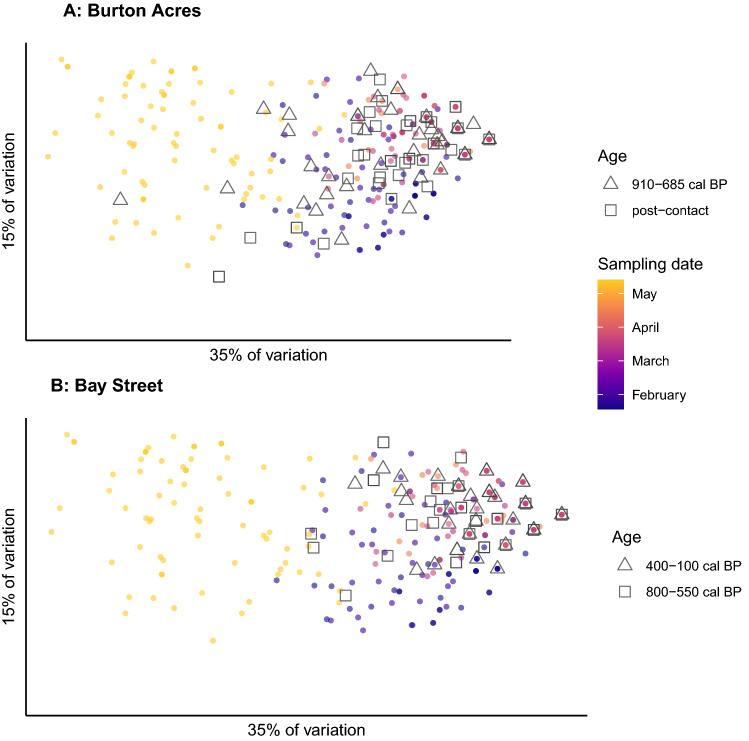
Principal Component Analyses of modern and ancient herring samples based on genetic variability at seven SNP loci. Modern samples are represented as points whose color indicates the date of sample collection (as in Supplemental Fig. 1). The ancient herring samples are represented by black triangles or squares, depending on their estimated age. (**A**) Comparison of archaeological herring from the Burton Acres Shell Midden to modern herring; (**B**) Comparison of archaeological herring from the Bay Street Shell Midden to modern herring.

In a PCA, most archaeological samples clustered with contemporary January–February and March–April spawners (Fig. 2). Pairwise *F*_*ST*_ between archaeological and contemporary sample collections ranged from − 0.002 to 0.416, and the archaeological collections were most divergent from contemporary May spawners. Pairwise *F*_*ST*_ between the two archaeological sites ranged from 0.003 to 0.046 (SI Data 4).

**Figure 3:**
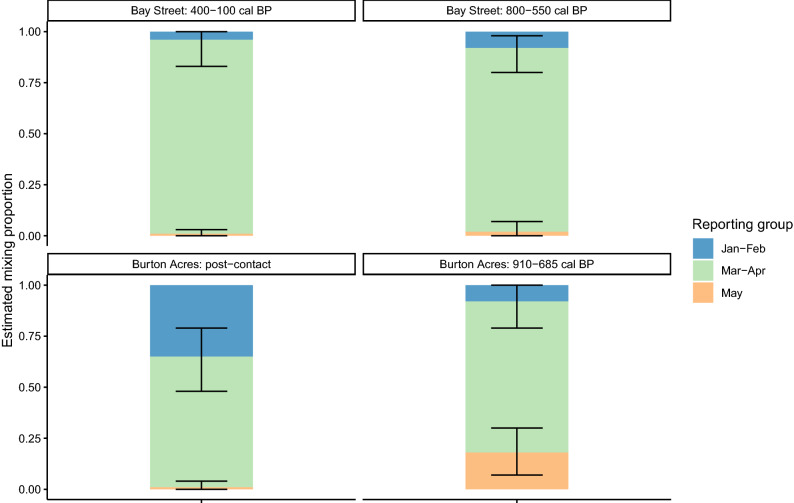
Results of mixed stock analysis showing the relative proportions of major spawning groups in archaeological herring samples. Estimated mixture proportions are displayed on y-axis and error bars indicate the 95% credible intervals. Different panels represent distinct archaeological sites and stratigraphic layers, while colors correspond to reporting groups.

A mixed stock analysis indicated that March–April spawners were abundant in both archaeological sites and across archaeological layers, and they made up a great proportion of herring deposited at the Bay Street Shell Midden (Fig. 3, Supplemental Fig. 5, and Supplemental Table 4). January–February spawners were rare in the Bay Street Shell Midden, but they were more common at the Burton Acres archaeological site. May spawners were absent from all samples except in the older archaeological layer from Burton Acres.

## Discussion

By analyzing archaeological fish assemblages with aDNA, we were able to reconstruct the population diversity of herring caught by Coast Salish people over multiple centuries. Our genetic data revealed that overall catches from central Puget Sound archaeological sites predominantly consisted of herring that spawned in winter and early spring (January–February and March–April spawners; Fig. 3). When comparing across archaeological sites, we observed differences in the composition of herring catches. At the Bay Street Shell Midden, people primarily harvested March–April spawners and this pattern was consistent over a period of hundreds of years. In contrast, Coast Salish people at the Burton Acres site used a portfolio of herring populations, composed of the three major spawn groups that are still observed in contemporary Puget Sound herring. The more recent assemblage from Burton Acres (post-contact) contained both January–February and March–April spawners, while a small number of May spawners was also detected in the older (910–685 cal BP) Burton Acres assemblage (Fig. 3).

Coast Salish occupants of the Burton Acres site benefitted from the ecological resource wave created by diverse spawning phenotypes. For example, phenological diversity in herring spawning behavior may have allowed people at Burton Acres to harvest herring and/or herring predators such as seals and salmonids in the nearshore environment over an extended period of time. Additionally, this prolonged spawn period would have enabled people to spread the labor of fishing and preserving herring and roe. By harvesting from multiple populations with different spawn times, Coast Salish people may have also ensured that no single population was overexploited. Finally, phenologically diverse spawn groups may have buffered fishing communities from variability in the abundance of any single population via portfolio effects^2^. Although the temporal resolution of our archaeological data is coarse, the overall abundance of herring bones is less variable through time at the genetically diverse Burton Acres Shell Midden than at the more homogenous Bay Street Shell Midden (Supplemental Fig. 3).

### Ecological and cultural context of herring harvests

Ancient faunal assemblages are the product of human foraging behaviors and dietary practices, as well as resource availability and taphonomic processes. Thus, there are multiple variables regarding the spatial scale of fishing, the relative abundance of herring populations, and/or the seasonality of herring harvest that may explain why specific spawn groups were highly abundant in the archaeological sites that we analyzed. We discuss different variables and their interactions below and place them in the context of available ecological and ethnographic information.

Regional analyses of archaeological sites in the Pacific Northwest^24,65^ indicate that species encountered in faunal assemblages are correlated with local habitats; for example, riverine sites typically contain abundant salmon bones while coastal sites are characterized by marine species such as rockfish, flatfish, cod, and sculpin. Pacific herring are relatively abundant throughout Puget Sound year-round^66,67^ but their reproductive behavior makes them easily accessible to coastal fishers and predators in the weeks and months prior to spawning, as adult fish aggregate to the nearshore environment to reproduce^68^. This pattern of local resource use is reflected in the archaeological record, as archaeological sites with a very high abundance (> 80%) of herring bones are spatially correlated with modern spawning locations, often co-occurring at distances less than 1 km, while sites with lower abundances of herring bones are more variable in regards to their distance to contemporary spawning locations^22^. These findings suggest that fishing for herring was concentrated around spawning grounds or nearby ‘pre-spawn holding areas’, locations where herring aggregate in the weeks leading up to reproduction. Additionally, there is ethnographic evidence that some of the herring harvest occurred during the spawning season: “*During the spawning season when large schools of herring and smelt crowded in to shore, the fish were dipped out with a loosely twined piece of matting…”*^28^.

Comparing the archaeological data to contemporary distributions of spawning herring can provide insights into the spatial extent and seasonality of fishing, as well as the long-term distribution of herring populations. Both archaeological sites analyzed here are proximate (within 10 km) to contemporary winter and early-spring spawning grounds (Fig. 1). In contrast, multidecadal spawn surveys^17^ show that the only May-spawning population reproduces at Cherry Point, a location that is approximately 170 km to the north of the sampled archaeological sites (Fig. 1). Genetic data indicate that contemporary herring populations exhibit seasonal and geographic fidelity to spawning areas across decades^18^, thus it is possible that the predominance of January–February and March–April spawners in central Puget Sound archaeological sites of different ages is the result of both (i) geographic and temporal stability in spawning activity and (ii) place-based fishing during the spawning season. To rigorously test this hypothesis, future research efforts should analyze archaeological specimens collected from locations used by contemporary May spawners and compare patterns of resource use over large geographic areas. This archaeological information could yield insights into the homing behavior of herring and help place documented extirpations of contemporary herring populations in the appropriate long-term ecological context.

Differences in the seasonal distribution and relative abundance of herring populations also likely contributed to the patterns observed in the archaeological record. In other words, it is possible that Coast Salish people in central Puget Sound primarily harvested January–February and March–April spawners because of these populations’ long residence times in the estuary and their localized abundance. It is important to consider this scenario, as it is documented that Coast Salish people also fished for herring outside of the spawning season^28^, and herring caught at different times of year might have served different purposes; for example, herring caught in winter may have been eaten fresh, while herring harvested at other times of year may have been dried^45^ or smoked^28^ for storage. There is limited contemporary information on the population-specific movements and distribution of Puget Sound herring outside of their reproductive season, but otolith microchemistry^69^ and persistent organochlorine pollutants^70^ demonstrate that contemporary Puget Sound herring spawning in winter and early spring have different chemical signatures from Cherry Point May-spawners. These results indicate that the May-spawning Cherry Point population migrates to offshore water to feed and spends relatively little time in the Puget Sound estuary. If these migratory behaviors were consistent through time, then Coast Salish people fishing in central Puget Sound would have predominately encountered January–February and March–April spawners when fishing locally, even if they were fishing outside of the spawning season. The small number of May spawners we detected in the older archaeological assemblage from Burton Acres could indicate that (i) this population was more widely distributed in the past or (ii) people traded herring captured from different locations in the Puget Sound.

The populations of herring preserved in the archaeological record could also reflect seasonal variability in the desirability of this resource. Pacific herring and its roe are calorie-rich and nutritious^71^, and these items may have been an especially welcome food source in the winter and early spring when other fresh foods were not easily accessible or in short supply. For example, here is an account of the seasonal importance of herring to the Puyallup and Nisqually people in central Puget Sound: “*Herring eggs were, also, eaten fresh with smoked salmon or, if the supply of smoked salmon were exhausted by the time herring eggs were available, they were eaten with sprouts*”^28^. Recent oral histories from the Haida First Nation in British Columbia also document this seasonal importance of herring: “*Along with the berries, the herring was the first from the ocean to be harvested. Our women would gather in their boats to harvest the K’aaw* [herring roe on kelp] *and celebrate another cycle of life”*^72^. Thus, the predominance of January–February and March–April spawners in our archaeological sites could have been driven by people’s foraging preferences, and indicate that herring were especially desirable during the winter and early spring, when other fresh foods were unavailable or difficult to obtain.

Although the temporal resolution of archaeological sites analyzed in this study is limited to centennial scales, our results demonstrate that human communities in central Puget Sound predominantly harvested winter and early-spring spawners over a period of several hundred years. This consistency in resource use is noteworthy given that our sampling period may have encompassed the transition between the climatic oscillations of the Medieval Warm Period (900–1200 CE, 800–1100 BP) and Little Ice Age (1300–1850 CE, 150–700 BP), which are associated with large changes in resource use in other coastal communities such as the Norse Greenlanders^73^ and New Zealand Maori^74^. Bioenergetic models indicate that warm oceanic regimes are associated with low recruitment and biomass in Pacific herring^75^, and water temperatures are estimated to have been ~ 0.9 °C warmer during the Medieval Warm Period than during the Little Ice Age in the Pacific Ocean^76^. Despite this potential climatic variability, our data suggest that winter and early spring spawners remained abundant enough that fishing communities in central Puget Sound could access them. This could be an indication that herring population diversity and/or traditional management and harvesting practices were resilient to past climatic oscillations. Stability in marine resource use across this time is also observed in other archaeological sites on the Northwest Coast^77,78^.

### Ancient population genetics and the quantification of past diversity

By genotyping a small number of SNPs with divergent allele frequencies in modern herring populations, we were able to assign ancient individuals to contemporary populations and estimate their reproductive timing. This approach allowed us to genotype large numbers of ancient individuals in a cost-effective manner and obtain sample sizes similar to those used in genetic studies of modern populations. The ability to analyze robust sample sizes reduces random sampling error and may lead to more accurate estimates of genetic diversity in ancient food resources.

A limitation of our study is that we could only distinguish between a small number of reporting groups, rather than individual contemporary spawning populations. Furthermore, because we used a small number of loci isolated from contemporary samples, we may have missed additional layers of genetic diversity such as the presence of distinct winter spawning populations or extinct populations that are not included in our contemporary genetic baseline. Finally, our analyses assume that allele frequencies at loci differentiating contemporary populations are stable over time, and were thus also correlated with spawn timing in ancient populations. One of the SNPs used in our analyses is within a gene (*SYNE2*) that is strongly correlated with spawn timing in both Atlantic^79^ and Pacific herring^18^, suggesting that this genomic region is involved in the regulation of reproduction. In addition, allele frequencies at this locus are very divergent between winter and spring spawning populations across the Pacific Northwest coast but temporally stable within spawning groups^18^. These lines of evidence suggest that allele frequency differences at this locus are maintained by natural selection and have been present for a long time.

At present, there is a trade-off between analyzing whole genomes for small numbers of samples and analyzing many samples at few loci. Although working with aDNA is technically challenging and ancient samples may be limited or scarce, developments in laboratory techniques, such as hybridization capture^80^, and the declining cost of sequencing will make the analysis of large numbers of ancient individuals and loci more common in the future^81^. This will not only facilitate genomic studies in well-studied charismatic species such as large mammals and hominids but may also allow for population genetic and ecological investigations in species that are currently important to human livelihoods and ecosystem processes.

## Conclusion

Ecological studies have established that resource waves benefit consumers by extending foraging opportunities^21^. For example, phenologically diverse herring populations are important to the diets of surf scoters^20^ and Chinook salmon^19^, and recent declines in the overall abundance of phenologically distinct herring populations in the Puget Sound have may negatively impacted the growth and survival of marine predators like salmon^19^.

By analyzing ancient DNA preserved in archaeological faunal assemblages, we were able to identify population-level genetic diversity in herring that sustained communities of Coast Salish people over a period of multiple centuries. Patterns of genetic diversity observed in the archaeological data are associated with phenologically distinct spawning groups in contemporary herring which form the basis of an ecological resource wave. Our results indicate that phenologically diverse herring stocks were important to the food systems of Coast Salish people in the Puget Sound, and that intraspecific biodiversity underpins the ecosystem services provided by healthy forage fish populations.

Our experimental approach is applicable to investigations of resource use in other systems, and it can be used to assess long-term trends in population diversity and establish ecological baselines for conservation. This type of information will help reveal interactions between human cultures and the ecological services provided by phenologically diverse resources. Such studies may also help identify the seasonality of archaeological site occupation with more precision. Furthermore, the long-term temporal context provided by archaeological data is especially valuable as climate change and other human activities lead to the synchronization of resource phenology and the disruption of food web dynamics^82^.

## Supplementary Information

Supplementary Information 1.

Supplementary Information 2.

## Data Availability

All sample metadata and genotyping data are included in the electronic supplemental material. Scripts are publicly available at a GitHub repository (https://github.com/EleniLPetrou/Ancient_DNA_Herring_TaqMan).
