# Supplementary material for: Ancient DNA reveals phenological diversity of Coast Salish herring harvests over multiple centuries
Source: Sci Rep. 2022 Aug 6;12:13512. doi: 10.1038/s41598-022-17656-4 (PMC9357025; doi:10.1038/s41598-022-17656-4)
Supplement: Supplementary file 2 — Supplementary Information 2. [file 41598_2022_17656_MOESM2_ESM.docx]

**Supplemental Materials**

## Supplemental methods and results

*Evaluation of locus panel for mixed stock analysis*

We assessed the predicted accuracy of mixed stock analysis by simulating multiple mixtures of known mixture proportions using herring samples collected from spawning herring. Mixtures were simulated with the *assess_reference_loo* function in *rubias* (simulated mixture size = 48 individuals; number of repetitions of mixture simulation and MCMC = 50), and estimated and simulated mixture proportions were compared. We also conducted “100% simulations”, where all simulated individuals in the mixture were generated from the allele frequency distribution of a single reference population. We analyzed these simulated data using three reporting groups (January-February spawners vs. March-April spawners vs. May spawners).

To avoid upward bias in the predicted accuracy of mixed stock analysis, we followed the recommendations of Anderson (2010) and empirically tested the accuracy of these seven loci for mixed stock analysis. This was accomplished by genotyping additional herring samples that were not part of the genetic baseline used to select the set of seven loci (double cross-validation *sensu* Anderson 2010). These additional samples of spawning herring (*N* = 119) were collected from three different locations belonging to the three different reporting groups in the Salish Sea: Squaxin Pass (January-February spawners), Elliot Bay (March-April spawners), and Cherry Point (May spawners).

When all sample collections in Supplemental Table 1 were included in the genetic baseline, we found that the correlation between simulated and estimated stock proportions was *r^2^* = 0.69 for January–February spawners, *r*^2^ = 0.62 for March–April spawners, and *r*^2^ = 0.88 for May spawners. Simulated mixtures originating from a single reporting group (also known as 100% simulations) resulted in mean estimates of mixture proportions ranging from 82 to 98% (Supplemental Table 2). We investigated discrepancies between the simulated and estimated mixture proportions in the 100% simulation scenarios and found that Jan-Feb spawners were misassigning primarily to the Mar-Apr group (16% misassignment; Supplemental Table 2) and to a lesser extent to the May reporting group (1% misassignment).We subsequently checked whether any of our January-February or March-April baseline populations consisted of population mixtures, using the RAD sequencing data published in Petrou et al. (2021). We found that one of the three populations in the Jan-Feb reporting group (Similk Bay 2015) clustered with both the Jan-Feb and Mar-Apr spawners in a PCA of individuals genotyped at over 4000 SNPs (Supplemental Figure 2). Unlike other sample collections, individuals from Similk Bay were split evenly between the Jan-Feb and Mar-Apr PCA clusters. The Similk Bay samples also had slightly more loci out of HWE (8%) than other populations (5% on average), which may be indicative of a population mixture (Wahlund effect).

Taken together, these results suggest that the Similk Bay 2015 collection may consist of a mixture of fish from both the January-February and March–April stocks. Spawn surveys indicate that spawning activity at Similk Bay can span the period from early February to late April (Sandell et al. 2019), so it is possible that this particular geographic location is used by multiple spawning groups of herring, and we accidentally sampled a mixed aggregate of populations there. In light of these results, we decided to remove the Similk Bay 2015 sample collection from the genetic baseline and repeated all simulations in *rubias* (100% simulations and mixtures of known mixture proportions).

Using this updated genetic baseline, we found that there was better agreement between the simulated and estimated mixture proportions. For example, the 100% simulations resulted in mean estimates of mixture proportions ranging from 92 to 98% (Supplemental Table 2) and misassignment was split more evenly between the different reporting groups (Supplemental Table 2). Additionally, the correlation between simulated and estimated stock proportions improved (*r^2^* = 0.85 for January–February spawners, *r*^2^ = 0.85 for March–April spawners, and *r*^2^ = 0.88 for May spawners). All subsequent analyses of archaeological samples were conducted using the updated genetic baseline (i.e., Similk Bay samples were excluded from downstream analyses).

## Supplemental Tables and Figures

**Supplemental Table 1.** The geographic location, sample size (*N*), sampling date, and mixed stock analysis reporting group for sample collections of contemporary herring that were collected from spawning grounds.

| **Location name** | ***N*** | **Latitude** | **Longitude** | **Date** | **Reporting group** |
| --- | --- | --- | --- | --- | --- |
| Squaxin Pass | 42 | 47.20 | -122.94 | 1/17/2007 | Jan-Feb |
| Port Orchard | 46 | 47.69 | -122.59 | 1/27/2014 | Jan-Feb |
| Similk Bay | 48 | 48.44 | -122.57 | 2/25/2015 | Jan-Feb |
| Port Gamble | 22 | 47.87 | -122.60 | 3/12/2014 | Mar-Apr |
| Quilcene Bay | 16 | 47.80 | -122.85 | 3/26/2014 | Mar-Apr |
| Gabriola Island | 45 | 49.16 | -123.74 | 3/19/2015 | Mar-Apr |
| Elliott Bay | 47 | 47.62 | -122.36 | 4/15/2015 | Mar-Apr |
| Cherry Point A | 34 | 48.93 | -122.80 | 5/12/2014 | May |
| Cherry Point B | 47 | 48.93 | -122.80 | 5/9/2016 | May |

**Supplemental Table 2.** Results of mixed stock analyses of simulated 100% mixtures for each of the three spawning groups. All samples were included in the first set of analyses, while Similk Bay was excluded from the second set.

| **All samples** | **Jan-Feb** | **Mar-April** | **May** |
| --- | --- | --- | --- |
| Jan-Feb | 82.77 | 15.78 | 1.44 |
| Mar-Apr | 3.71 | 95.79 | 0.50 |
| May | 0.73 | 0.96 | 98.31 |
| **Without Similk Bay** |  |  |  |
| Jan-Feb | 91.68 | 6.07 | 2.25 |
| Mar-Apr | 2.49 | 96.94 | 0.57 |
| May | 0.52 | 1.08 | 98.40 |

**Supplemental Table 3.** Population genetic summary statistics calculated for the archaeological samples using seven DNA loci: Expected (*Hs*) and observed (*Ho*) heterozygosities, estimates of *F_IS,_* and p-values for tests of Hardy-Weinberg Equilibrium (*P*).

|  | **Sample collection** | **Burton Acres** | **Burton Acres** | **Bay Street** | **Bay Street** |
| --- | --- | --- | --- | --- | --- |
|  | **Temporal layer** | post-contact | 910-685 cal BP | 400-100 cal BP | 800-550 cal BP |
| **Locus_30660** | ***N*** | 44 | 36 | 41 | 45 |
|  | ***H_o_*** | 0.182 | 0.111 | 0.317 | 0.200 |
|  | ***H_s_*** | 0.204 | 0.354 | 0.302 | 0.338 |
|  | ***F_IS_*** | 0.109 | 0.686 | -0.048 | 0.409 |
|  | ***P*** | 0.437 | **0.000*** | 1.000 | **0.013** |
| **Locus_31014** | ***N*** | 44 | 39 | 41 | 46 |
|  | ***H_o_*** | 0.250 | 0.231 | 0.220 | 0.109 |
|  | ***H_s_*** | 0.221 | 0.347 | 0.235 | 0.143 |
|  | ***F_IS_*** | -0.132 | 0.335 | 0.067 | 0.237 |
|  | ***P*** | 1.000 | 0.054 | 0.538 | 0.218 |
| **Locus_519** | ***N*** | 44 | 39 | 41 | 46 |
|  | ***H_o_*** | 0.091 | 0.205 | 0.073 | 0.044 |
|  | ***H_s_*** | 0.273 | 0.265 | 0.117 | 0.085 |
|  | ***F_IS_*** | 0.667 | 0.225 | 0.372 | 0.486 |
|  | ***P*** | **0.000*** | 0.197 | 0.121 | 0.066 |
| **Locus_6354** | ***N*** | 45 | 37 | 41 | 46 |
|  | ***H_o_*** | 0.444 | 0.324 | 0.244 | 0.500 |
|  | ***H_s_*** | 0.504 | 0.500 | 0.399 | 0.486 |
|  | ***F_IS_*** | 0.118 | 0.351 | 0.389 | -0.029 |
|  | ***P*** | 0.550 | **0.046*** | **0.019*** | 1.000 |
| **Locus_725** | ***N*** | 40 | 30 | 41 | 44 |
|  | ***H_o_*** | 0.175 | 0.067 | 0.049 | 0.296 |
|  | ***H_s_*** | 0.369 | 0.238 | 0.095 | 0.343 |
|  | ***F_IS_*** | 0.526 | 0.720 | 0.484 | 0.139 |
|  | ***P*** | **0.002*** | **0.002*** | 0.074 | 0.384 |
| **Locus_8468** | ***N*** | 45 | 36 | 41 | 46 |
|  | ***H_o_*** | 0.378 | 0.222 | 0.390 | 0.370 |
|  | ***H_s_*** | 0.442 | 0.505 | 0.456 | 0.495 |
|  | ***F_IS_*** | 0.146 | 0.560 | 0.144 | 0.254 |
|  | ***P*** | 0.492 | **0.002*** | 0.486 | 0.129 |
| **Locus_24510** | ***N*** | 45 | 39 | 41 | 46 |
|  | ***H_o_*** | 0.133 | 0.026 | 0.122 | 0.000 |
|  | ***H_s_*** | 0.126 | 0.026 | 0.116 | 0.044 |
|  | ***F_IS_*** | -0.060 | 0.000 | -0.053 | 1.000 |
|  | ***P*** | 1.000 | Not calculated | 1.000 | **0.011** |

**Supplemental Table 4.** Results of mixed stock analysis, showing the mean estimated proportion (mean) and the 5^th^ - 95^th^ Credible Intervals (CI) for archaeological herring samples from the same site and chronological unit.

| **Archaeological Site** | **Chronological layer** | **Jan-Feb** | **Jan-Feb** | **Mar-Apr** | **Mar-Apr** | **May** | **May** |
| --- | --- | --- | --- | --- | --- | --- | --- |
|  |  | **mean** | **CI** | **mean** | **CI** | **mean** | **CI** |
| Bay Street Shell Midden | 400-100 cal BP | 0.04 | 0.00-0.17 | 0.95 | 0.83-1.00 | 0.01 | 0.00-0.03 |
| Bay Street Shell Midden | 800-550 cal BP | 0.08 | 0.01-0.18 | 0.90 | 0.79-0.98 | 0.02 | 0.00-0.07 |
| Burton Acres Shell Midden | Post-contact | 0.35 | 0.20-0.52 | 0.64 | 0.48-0.79 | 0.01 | 0.00-0.04 |
| Burton Acres Shell Midden | 910-685 cal BP | 0.08 | 0.01-0.21 | 0.74 | 0.58-0.88 | 0.18 | 0.07-0.30 |

**
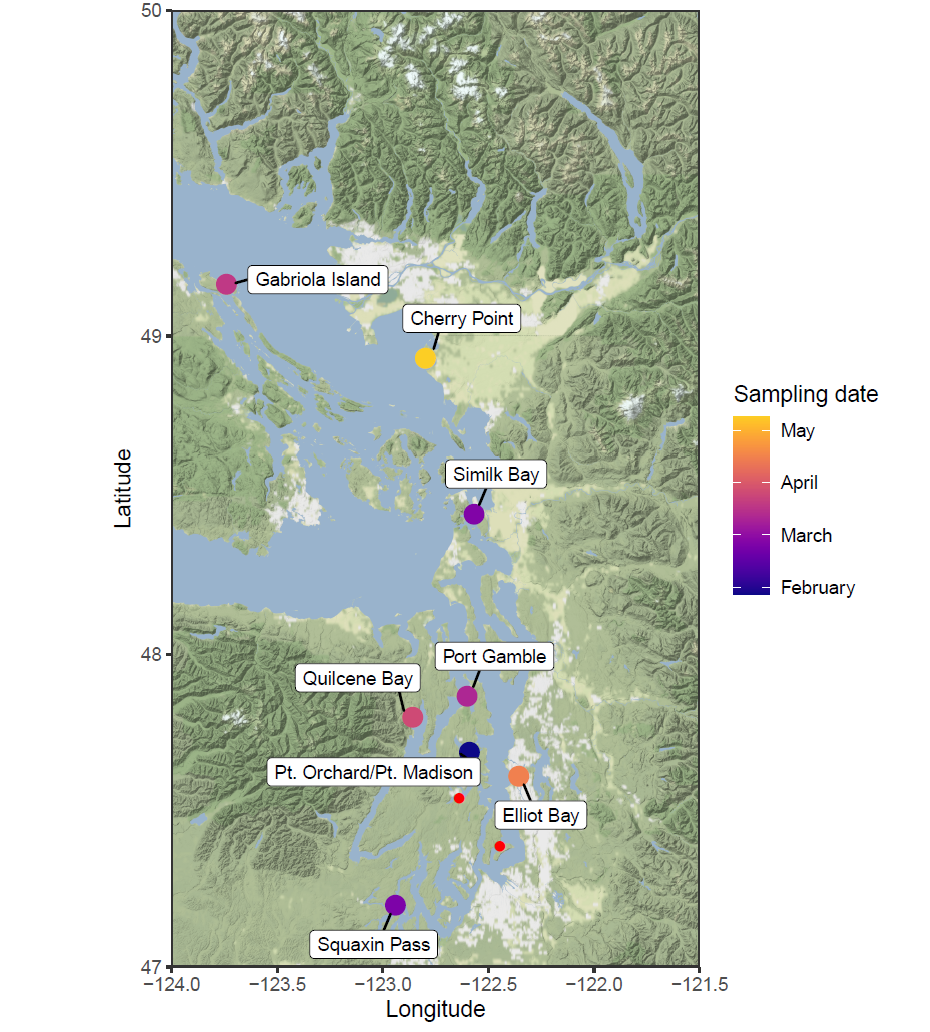
**

**Supplemental Figure 1.** Sampling locations of contemporary herring populations. The color of each circle depicts the date of sampling an aggregation of spawning herring or fertilized herring eggs. The two archaeological sites are depicted by red points. The map was created using the R package *ggmap* v. 3.0.0 using map tiles by Stamen Design, under CC BY 3.0. Data by OpenStreetMap, under the Open Data Commons Open Database License (ODbL) by the OpenStreetMap Foundation (https://www.openstreetmap.org/copyright).


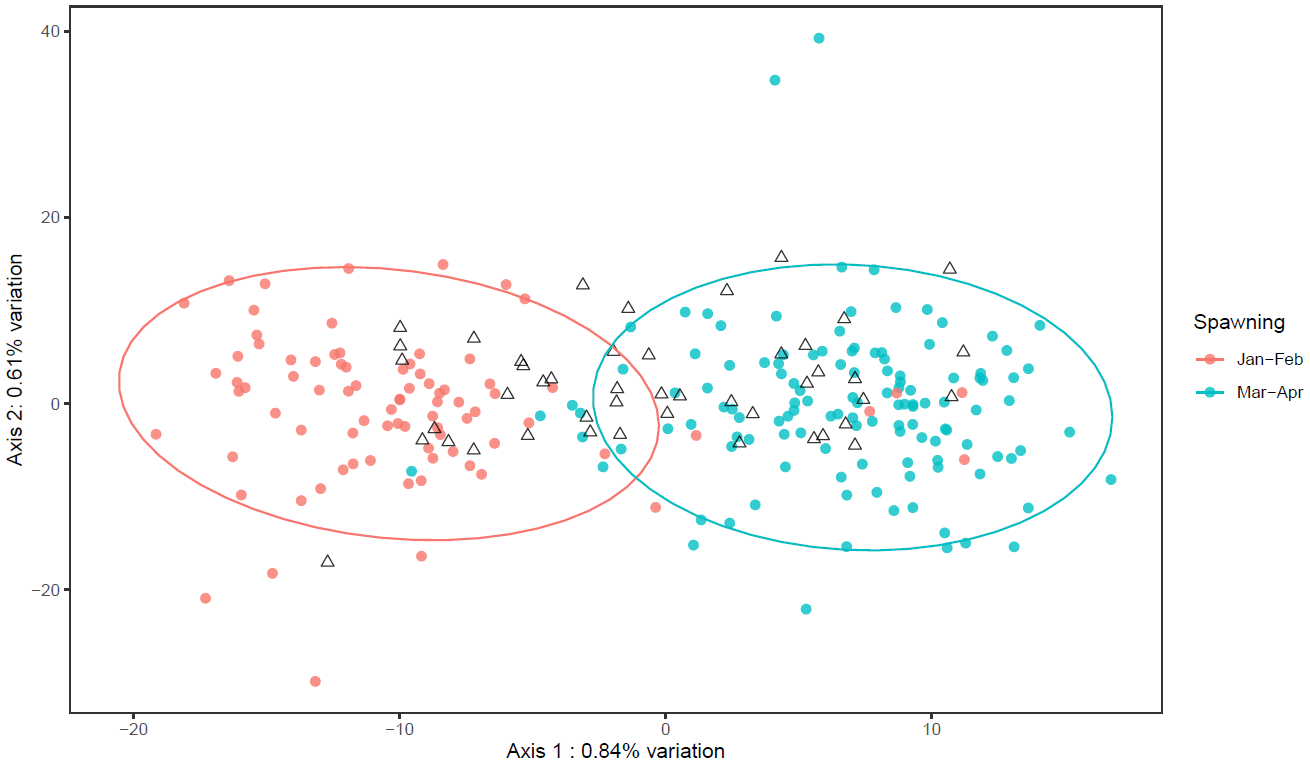
**Supplemental Figure 2.** PCA of baseline samples (contemporary Jan-Feb and Mar-Apr spawners listed in Supplemental Table 1) genotyped using 4,888 SNPs that have been pruned for linkage disequilibrium (data published in Petrou et al. 2021). The Similk Bay 2015 samples are shown by the open black triangles.

**
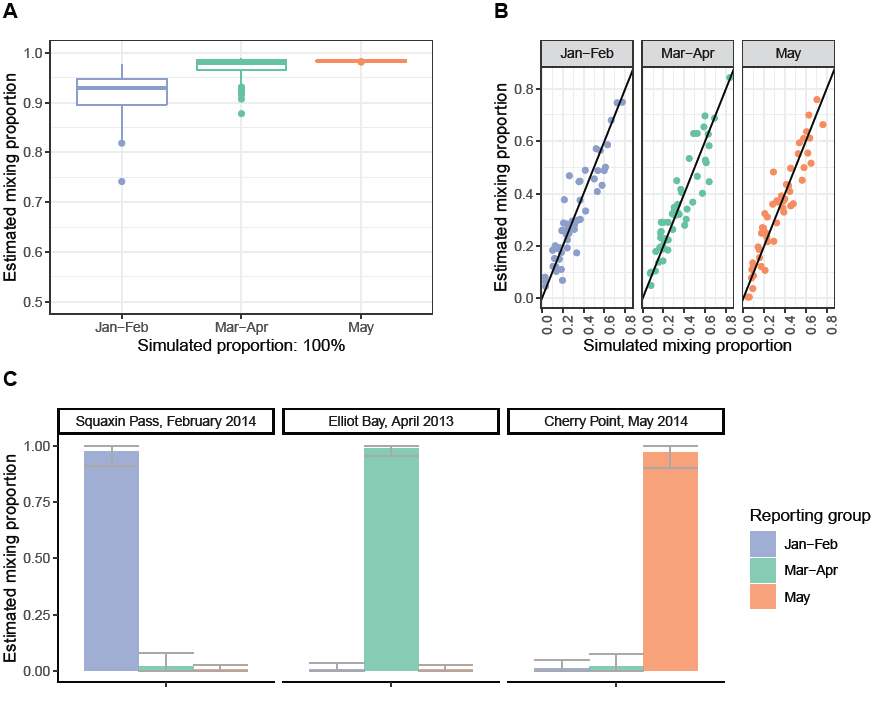
**

**Supplemental Figure 3.** Predicted accuracy of mixed stock analysis using seven loci and three reporting groups (represented by different colors). Data from Chamberlin et al.(2021), excluding samples from Similk Bay 2015. A) Results of 100% simulations using simulated data. B) Correlation between the estimated and true mixture proportions using simulated data; the diagonal line indicates expectations for perfect assignment. C) Evaluation of mixed stock analysis using additional samples (not used for locus discovery) collected from Squaxin Pass, Elliot Bay, and Cherry Point. Error bars indicate the 5th-95th credible intervals around the mean estimated proportion of individuals assigned to a particular reporting group.

**
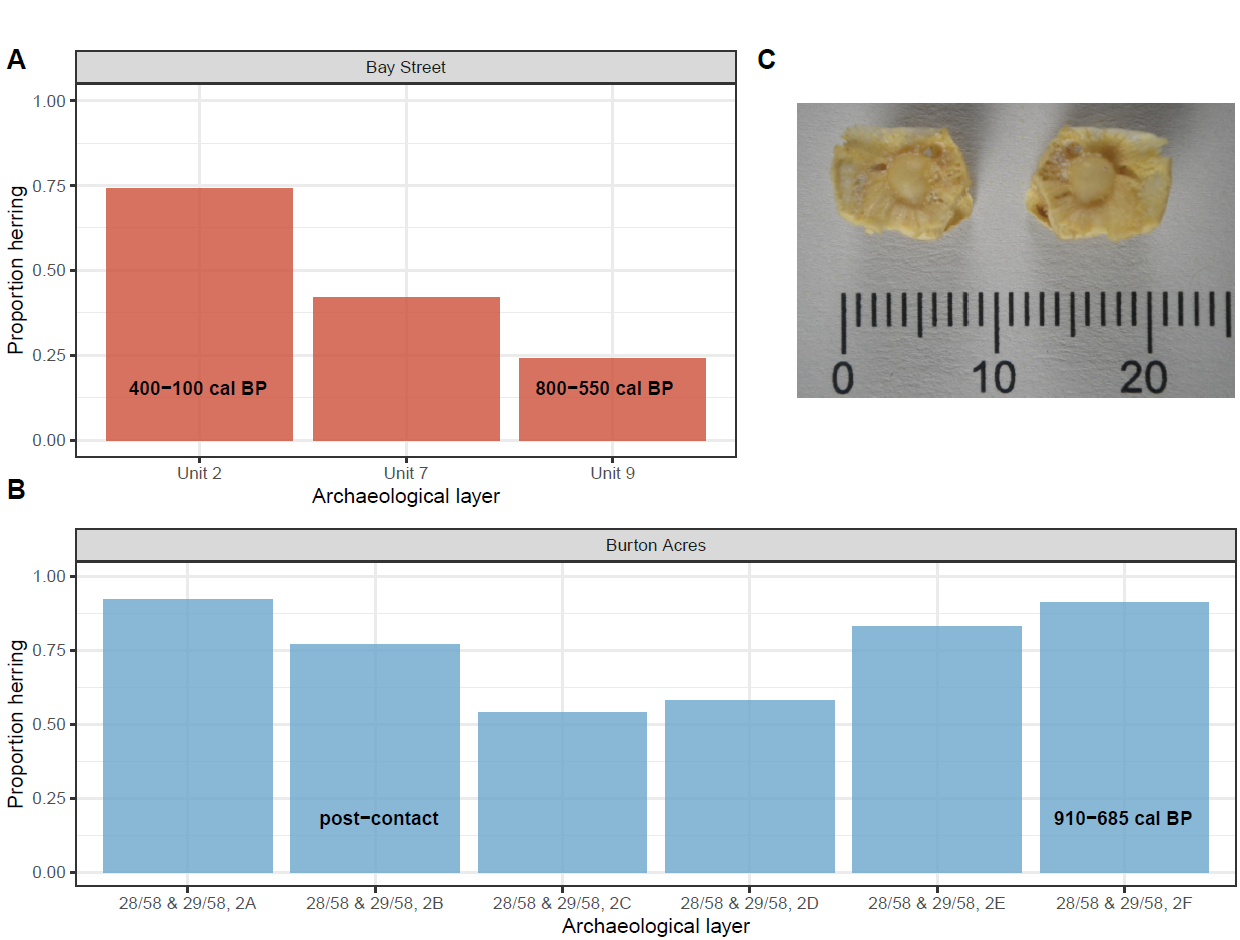
**

**Supplemental Figure 4.** Proportion of herring bones relative to other fish bones found at each archaeological site. Archaeological layers are labelled with the name provided in the original publication. Layers sampled for aDNA in this study are also labelled with the estimated radiocarbon dates (cal BP). A) Results from Bay Street Shell Midden (data from Lewarch et al.(Lewarch et al. 2002), B) results from Burton Acres Shell Midden (data from Kopperl (Kopperl 2001)), and C) size of herring prootic bones (mm).

**
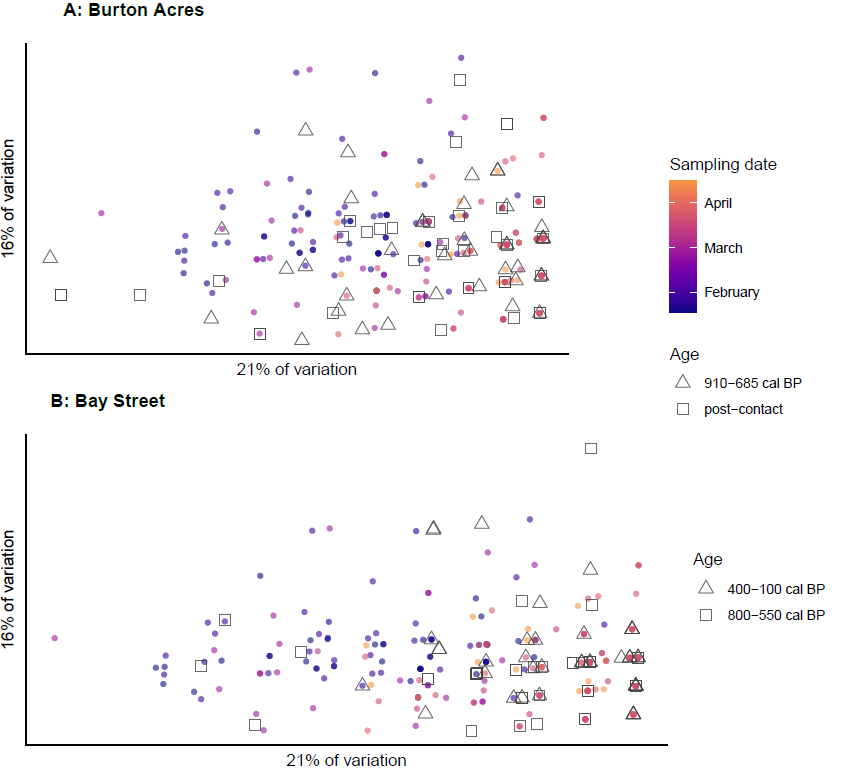
**

**Supplemental Figure 5.** PCA of modern and ancient herring samples based on genetic variability at seven SNP loci and excluding May-spawners from the analysis. Modern samples are represented as points whose color indicates the date of sample collection. The ancient herring samples are represented by black triangles or squares, depending on their estimated age. A) Comparison of archaeological herring from the Burton Acres Shell Midden to modern herring; B) Comparison of archaeological herring from the Bay Street Shell Midden to modern herring.

**Literature Cited**

Anderson, E. C. 2010. Assessing the power of informative subsets of loci for population assignment: standard methods are upwardly biased. Molecular Ecology Resources **10**:701-710.

Chamberlin, J., E. Petrou, W. Duguid, R. Barsh, F. Juanes, J. Qualley, and L. Hauser. 2021. Phenological diversity of a prey species supports life-stage specific foraging opportunity for a mobile consumer. ICES Journal of Marine Science **78**:3089-3100.

Kopperl, R. E. 2001. Herring use in southern Puget Sound: analysis of fish remains at 45-KI-437  Northwest Anthropological Research Notes **35**:1-20.

Lewarch, D. E., L. A. Forsman, S. K. Kramer, L. R. Murphy, L. L. Larson, D. R. Iversen, and A. E. Dugas. 2002. Data recovery excavations at the Bay Street Shell Midden (45KP115), Kitsap County, Washington. Larson Anthropological Archaeological Services Limited.

Petrou, E. L., A. P. Fuentes-Pardo, L. A. Rogers, M. Orobko, C. Tarpey, I. Jiménez-Hidalgo, M. L. Moss, D. Yang, T. J. Pitcher, T. Sandell, D. Lowry, D. E. Ruzzante, and L. Hauser. 2021. Functional genetic diversity in an exploited marine species and its relevance to fisheries management. Proceedings of the Royal Society B: Biological Sciences **288**:20202398.

Sandell, T., A. Lindquist, P. Dionne, and D. Lowry. 2019. 2016 Washington State herring stock status report. Washington Department of Fish and Wildlife.
